# Remote sensing observation of annual dust cycles and possible causality of Kawasaki disease outbreaks in Japan

**DOI:** 10.21542/gcsp.2017.22

**Published:** 2017-10-31

**Authors:** Hesham El-Askary, Nick LaHaye, Erik Linstead, William A. Sprigg, Magdi Yacoub

**Affiliations:** 1 Schmid College of Science and Technology, Chapman University, Orange, CA, USA; 2 Center of Excellence in Earth Systems Modeling & Observations, Chapman University, Orange, CA, USA; 3 Department of Environmental Sciences, Faculty of Science, Alexandria University, Moharem Bek, Alexandria, Egypt; 4 Jet Propulsion Laboratory, California Institute of Technology, CA, USA; 5 Institute for Atmospheric Physics, The University of Arizona, Tucson, AZ, USA; 6 Faculty of Medicine, National Heart & Lung Institute, Imperial College of London

## Abstract

Kawasaki disease (KD) is a rare vascular disease that, if left untreated, can result in irreparable cardiac damage in children. While the symptoms of KD are well-known, as are best practices for treatment, the etiology of the disease and the factors contributing to KD outbreaks remain puzzling to both medical practitioners and scientists alike. Recently, a fungus known as *Candida,* originating in the farmlands of China, has been blamed for outbreaks in China and Japan, with the hypothesis that it can be transported over long ranges via different wind mechanisms. This paper provides evidence to understand the transport mechanisms of dust at different geographic locations and the cause of the annual spike of KD in Japan.

*Candida* is carried along with many other dusts, particles or aerosols, of various sizes in major seasonal wind currents. The evidence is based upon particle categorization using the Moderate Resolution Imaging Spectrometer (MODIS) Aerosol Optical Depth (AOD), Fine Mode Fraction (FMF) and Ångström Exponent (AE), the Cloud-Aerosol Lidar and Infrared Pathfinder Satellite Observation (CALIPSO) attenuated backscatter and aerosol subtype, and the Aerosol Robotic Network’s (AERONET) derived volume concentration.

We found that seasonality associated with aerosol size distribution at different geographic locations plays a role in identifying dominant abundance at each location. Knowing the typical size of the *Candida* fungus, and analyzing aerosol characteristics using AERONET data reveals possible particle transport association with KD events at different locations. Thus, understanding transport mechanisms and accurate identification of aerosol sources is important in order to understand possible triggers to outbreaks of KD. This work provides future opportunities to leverage machine learning, including state-of-the-art deep architectures, to build predictive models of KD outbreaks, with the ultimate goal of early forecasting and intervention within a nascent global health early-warning system.

## Introduction

In some incidences, heart-related diseases have been related to environmentally driven factors, potentially compounded by climate change and reduced resources^1^. For instance, about 100 years ago, Rheumatic Fever (RF)/Rheumatic Heart Disease (RHD) was believed to be an ailment of “temperate climate”^2^. RHD cases were reported from the plains of India, and are less common than reported in colder climates^3^. On the other hand, the absence of haemolytic streptococcal infections and low prevalence of RF/RHD in the tropics has also been reported^4^. Environmental factors can play a role in disease outbreaks because some infectious agents had the potential to survive low temperature (<−40°C), low relative humidity (<30% in the western north Pacific), and high ultraviolet exposure^5^. This was clearly documented in the case of African dust particles harboring *Aspergillus sydowii*, responsible for diseases affecting coral in the Caribbean^5^. Moreover, using a PM10 and PM2.5 categorical model, it was shown that relative risks of cardiovascular diseases (rheumatic heart disease, hypertension, ischemic cardiovascular diseases, arrhythmia, congestive heart failure) were increased with intensity of dust and sand events (from normal clean day, to light contaminated day to blowing sands day, and to dust storm day) in a dose-related manner^6–8^.

Kawasaki disease is seen as one of the main drivers for acquired heart diseases in children in different developed parts of the world^9–12^. KD is a disease characterized by seasonal outbreaks that match other seasonal earth system processes that can induce, or at least be a vital player, in the outspread^13–17^.

With the continuous rise in global temperature anomalies, more extreme weather-related events have increased either in magnitude or in frequency^18^. Among these are globally occurring intense heat waves, associated prolonged droughts, and more frequent dust storms^18^. Such extremes promote dry, loose soil, which leads to more aerosols, loaded with spores, pollens, fungus, and microbes that might be present in that soil, to be blown into the air.

It has long been assumed that the sun’s ultraviolet (UV) radiation will kill any airborne microbes^19^, yet we believe that fine dust particles shield these agents by scattering the UV radiation, and hence provide a safe environment while being transported over great distances. It was found that concurrent outbreaks of KD in Japan, the mainland US, and Hawaii might stem from processes involving weather or climate variations, revealing possible connections to atmospheric circulation patterns^13, 14^.

In this work, the authors performed a time series analysis of KD patients in three geographically distant regions suggesting that the agent responsible for KD is transported through broad scale wind currents - implying that its causes are not exclusively genetic^20^.

The seasonal wind patterns associated with increases in KD cases are, specifically, a northwesterly trajectory from the mainland of central/eastern Asia sweeping Japan, as well as a zonal wind pattern that traverses the north Pacific, spanning from Japan to Hawaii and ultimately reaching the mainland of the United States^16, 17^.

According to clinical analyses, infectious disease specialists, and molecular microbiologists, the triggers to KD outbreaks are still debatable^15–17^. The causal agent of KD remains unknown after more than 40 years of intensive research^13, 14^. Hence, environmental triggers may, to a greater or lesser extent, contribute to the anomalous KD outbreaks in areas prone to dust outbreaks, or even to areas that experience long range transportation of aerosols^13, 14^. These aerosols might contain a microbiome that includes the KD agent.

In this paper we demonstrate that the aforementioned wind patterns are also associated, on an annual basis, with known dusty seasons and fine aerosol outbreak that peak, and are followed by a drop in dust outbreaks that interestingly match an observed decline in KD outbreaks reported in Japan^11^. Here, we utilize satellite driven parameters, namely, Aerosol Optical Depth (AOD), Fine Mode Fractions (FMF), Ångström exponent (AE) and particle volume concentration parameters to present the persistent annual cycle of fine dust occurrence when KD outbreaks are reported in Japan^11^. Anomalies in KD observations often exhibited “a coherent pattern” across the Japanese archipelago. This finding can guide physicians and scientists in identifying the causality of the KD outbreaks and any association with dusty season. This will lead satellite based observations in conjunction with predictive models to forecast critical aerosol outbreaks, their sources and their transport on hemispheric wind that could, in turn, alert clinicians to upcoming periods of regional KD risk. As such, efforts to isolate the causative agent of KD should focus on the microbiology of aerosols. This data-rich domain can allow for a forecasting system based on deep learning and other machine learning techniques applied to aerosol-related parameterizations^21^.

## Aerosol loading and long-range transport

Aerosol deposition from dust storms has increased in recent history. A changing climate along with poor management of Earth’s dry land are concerns that contribute to surface vegetation reduction, which increases the source of dust entrained in winds off from deserts and their fringes^22^. A dust storm can transport large amounts of sand, silt, dust, and other fine aerosol-particles, often unexpectedly, over long distances affecting land and ocean biota alike, locally and globally^23^. The Sahara Desert and arid land around the Arabian Peninsula are principle sources of sand and dust, with some contributions from Iran, Pakistan and India^22^.

Across Asia, dust sources of the Gobi and Taklimakan Deserts affect the Central and Eastern Asia air quality, especially affecting environments in China and Mongolia^24^. Asia’s significant storms deposit dust over Korea, Japan, and across the Pacific to influence the environments of Western America^25^.

Over the last decade, aerosols have been studied on different scales, regionally and globally, using satellite-based observations. These studies have been important in shedding light on the ultimate connections of windblown dust to the Earth’s global climate system^26–28^. Aerosols at different localities originate from a variety of sources, whether natural (e.g., sea salts, desert dust) or anthropogenic (local sources) due to increasing urbanization and industrialization. They all are capable of, long-range transport across the globe^29–35^.

Clearly identifying the correct sources of aerosols is important for attribution and amelioration of climate change and for public health risk reduction^36^. Dust models complemented with satellite observations fill gaps in conventional approaches to air quality and disease surveillance, as in the case of Valley Fever associations with the American Haboob of Arizona in 2011^37^. Such studies aid understanding of the atmospheric life cycle of cocci spores with simulated spore emission, transport, and deposition^37, 38^.

Air masses loaded with dust have been well documented in their ability to transport fungi causing Valley Fever and KD outbreaks when inhaled. A soil-dwelling fungus (*Coccidioides immitis* and *Coccidioides posadasii*) and its arthroconidia, fragments approximately 2–5 µm in length, cause Valley Fever (coccidioidomycosis) when inhaled and can be fatal^39–43^.

Learning from the Valley Fever case, let us examine potential transport of Candida fungus from Asia. In spring, the Aleutian atmospheric pressure low in the North Pacific weakens and a strong high pressure center develops in the subtropical North Pacific. At the same time, typically strong northwesterly winds are interrupted in Japan, and the wind’s path across the North Pacific is redirected to higher latitudes. This atmospheric path is much weaker and shifts northward in spring and summer. This springtime interruption coincides with the seasonal decline in KD cases in both San Diego and Hawaii^13, 14^.

**Figure 1.**
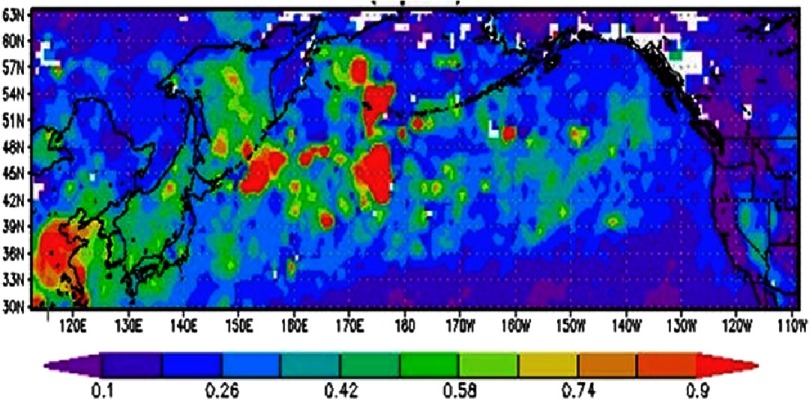
MODIS Aerosol Optical Depth showing the long range transport of dust from Asia to North America.

Figure 1 shows the long-range transport of dust from Asia across the Pacific Ocean towards North America. Data are obtained from the Moderate Resolution Imaging Spectroradiometer (MODIS), aboard the NASA Terra satellite. MODIS provides a monthly aerosol product, AOD, which yields information about aerosol characteristics in the field of view. AOD is a measure of the opaqueness of air, with high values indicating poor visibility. MODIS sees a 2330 km wide swath as it passes over both land^44^ and ocean^45^. The instrument provides daily global coverage, albeit with narrow gaps between swaths in the tropical region, of several validated aerosol products, including AOD, AE and FMF that will be used in the present work^46, 47^.

## Annual dust activity during recorded KD outbreaks in Japan

**Figure 2.**
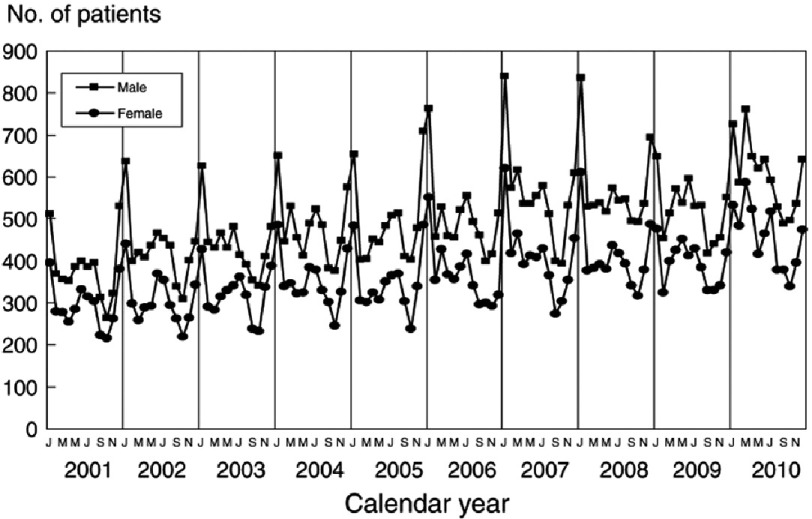
Seasonal Trends of Kawasaki Disease using hospital data (2001–2010)^12^.

**Figure 3.**
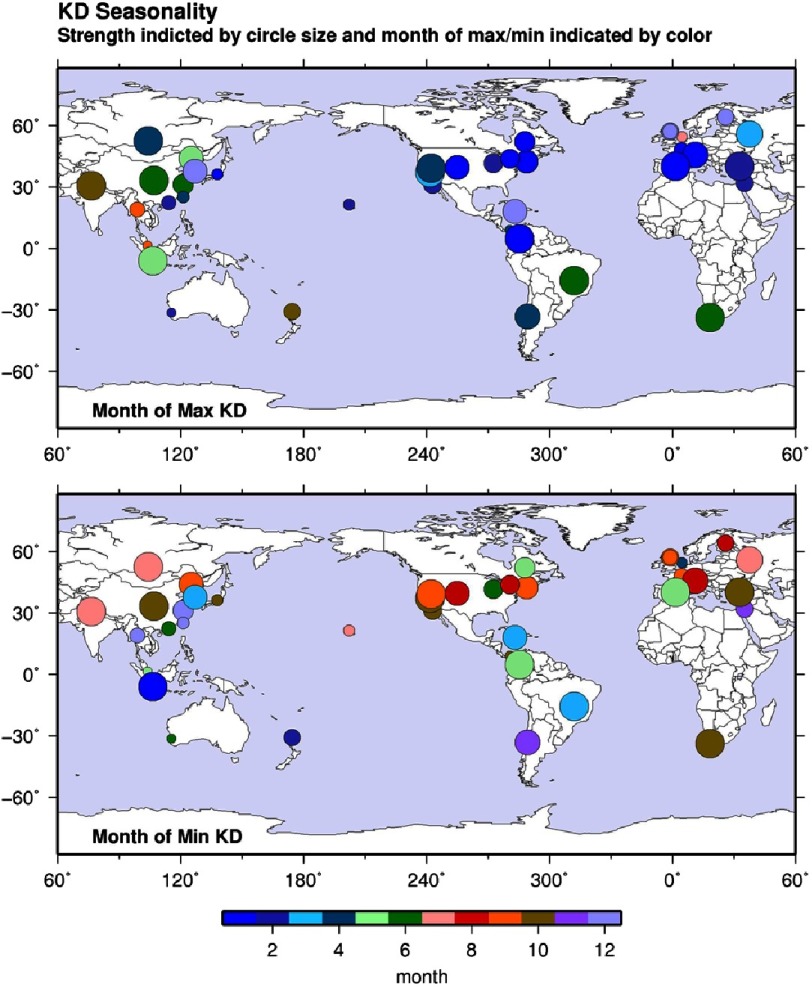
Maximum (top) and minimum (bottom) months of Kawasaki disease outbreaks from 1970–2012. Strength indicated by circle size and month of max/min indicated by colour. Reproduced under a CC-BY licence from [15].

The seasonal trends of KD patients have been explored from January 2001 to November 2010 (Figure 2)^12^. An overall upward trend in the number of patients, with a clear spike during the winter season, is followed by a smaller spike in summer, spring and fall. This behavior agrees with the reported maximum and minimum outbreak months in certain regions globally (Figure 3)^15^.

Air masses loaded with dust help transport different kinds of microbes and fungi. Accordingly, we believe that the long-range transported dust has contributed to KD outbreaks (Figure 2). Thus, we explore dust occurrences using the FMF and AE and their degree of correlation with KD outbreaks over Eastern China and Japan. We do so on an annual basis, whenever clinical data of KD outbreaks have been reported^12^. AE is derived from the ratio between *ln*(τ_1_∕τ_2_) and *ln*(λ_2_∕λ_1_), for given spectral band wavelengths λ_1_ and λ_2_, and their corresponding AODs τ_1_ and τ_2_^48^. It describes the mode, the most common, aerosol size distribution, differentiating between fine and coarse particles size. Dust particles, especially those with the ability to transport long distances, have a mid-range AE within a range of [0, 4]. On the other hand, the FMF is the fraction of the AOD contributed by fine aerosols (effective radius 0.1–0.25 µm)^46^. It shows the proportion of smaller particles of atmospheric aerosols to the total. These parameters, in combination with others, help us track dust events over time, as the signature of the fine mode particles can be seen as they travel or dissipate.

In the present work, the FMF and AE are plotted as Hovmoller diagrams (Figures 5, 6 and 7), a 2-dimensional contour plot that allows for the representation of 3 dimensions: time, space, and the value to be plotted. Hovmoller plots visualize a value’s distribution over time and 1 dimension of space. Because of this, we average data over all latitudes included in this study. The initial Hovmoller plot of the FMF shows the fine particle association with the reported KD outbreak during the time period that ranges from November 01, 2007 to February 01, 2009. However, KD reports have been extended to allow analysis for the period (2000–2010)^12^, hence, a longer time period analysis will be discussed later in this work.

**Figure 4.**
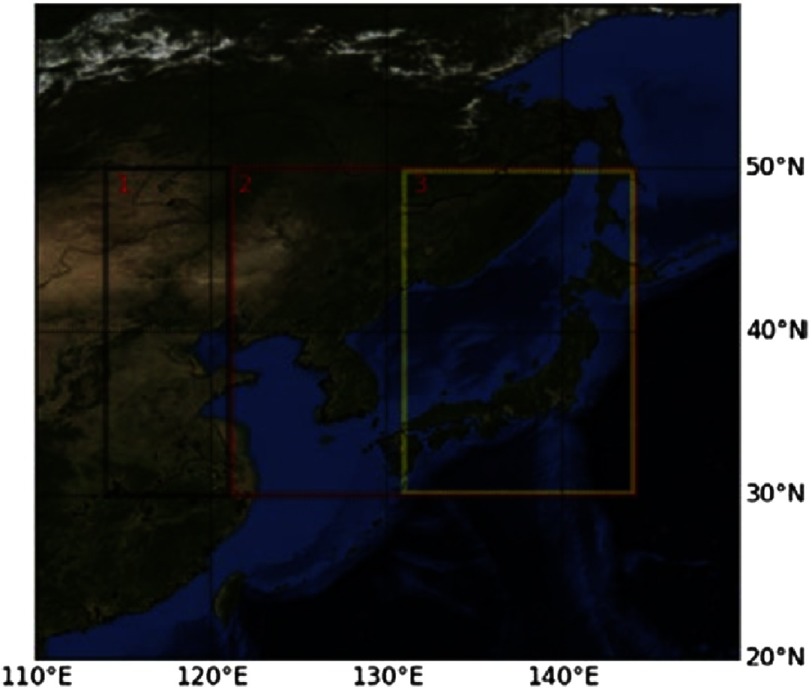
The regions under investigation in this study.

**Figure 5.**
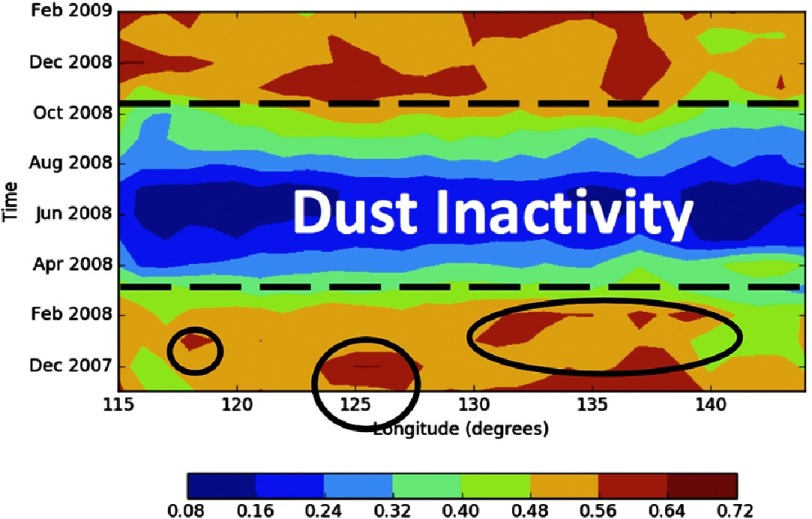
The FMF over land and ocean for the entire study region.

**Figure 6.**
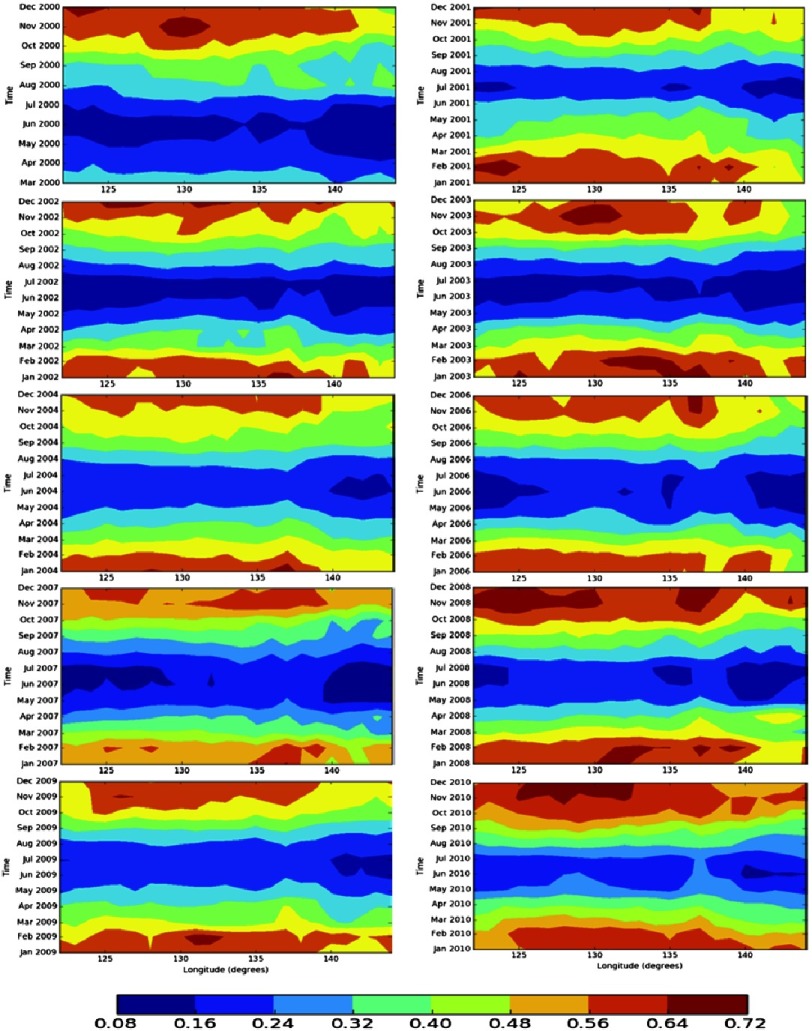
The FMF annual cycle over the study region (2000–2010) matching with the KD outbreaks reported by the hospital data shown in Figure 2^12^.

**Figure 7.**
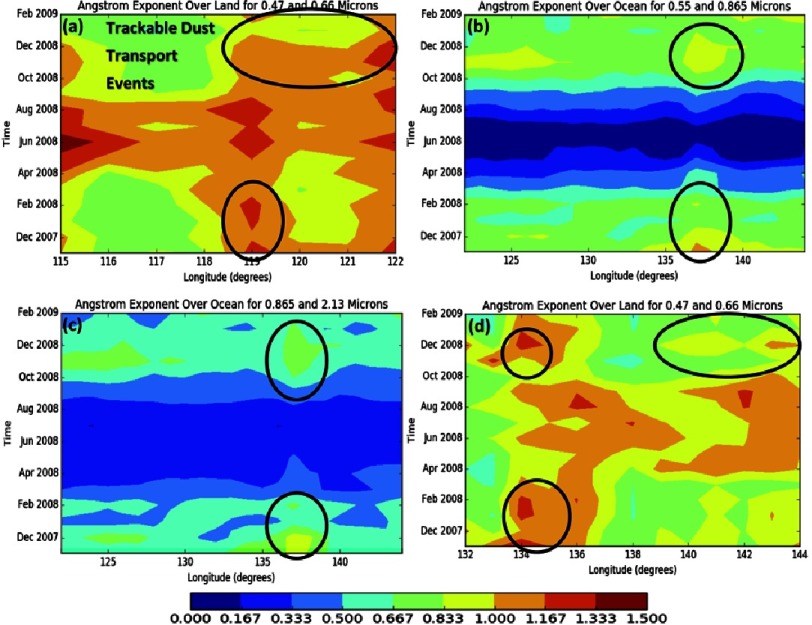
The Angstrom Exponent over land region 1 (Top Left), ocean (Top Right and Bottom Left), and land region 2 (Bottom Right).

To compensate for the fact that MODIS uses different algorithms and reflective bands over land and ocean, the area of study was divided into different regions (Figure 4). The black box, labeled 1, shows the high land region in China assumed to be the fungus source. The red box, labeled 2, shows where ocean values are plotted, and the yellow box, labeled 3, shows land regions in Japan. It should be noted that ocean values are also plotted in the region overlapping the yellow region.

The clear seasonal anomaly of dust attributed to the long-range transport of small particles can be seen in Figure 5. It shows the monthly FMF over the entire region, covering both land and ocean, under investigation where KD outbreaks were observed and previously reported during the period (2007–2009)^12^. In the bottom left corner a small peak in the FMF is observed followed by a much larger peak later in December 2007, centered at 125 degrees. Another peak begins in early January, followed by 2 more peaks. These peaks have been circled and they show large collections of fine mode particles that are moving from China to Japan. This will be further investigated using the AE, yet the FMF shows the times when particle transport is greatest, with a signature that denotes dust events in the winter, from November to the end of January. This is very much in agreement with the aforementioned seasonality of KD in this region^12, 15^.

These peaks are separated by a zone, or time period, of low dust activity, shown as a blue band between the winter seasons high activity (Figure 5). It is noteworthy that the number of KD patients recorded during the dust-inactive period dropped dramatically (Figure 2)^12^. Another period of dust activity appears late in 2008 and early 2009, and matches with a new spike in the number of KD patients. This analysis is extended further to cover the whole time period where an annual cycle in the FMF dust activity has been observed over the region. This, too, correlates with the annual rise and fall of the KD cases^12^ (Figure 6).

This analysis has been extended to look into the AE characteristics of aerosols to confirm that fine dust particles dominate during the reported KD outbreaks (Figures 7a, 7b, 7c & 7d).

The extinction AE (α_ext_) is calculated using the measured aerosol optical properties and AOD spectral dependence (τ_ext_) with wavelength (λ)^50^.

(1) \begin{eqnarray*}{\mathrm{\alpha }}_{\mathrm{ext}}=-\text{d}~\ln \nolimits [{\mathrm{\tau }}_{\mathrm{ext}}(\mathrm{\lambda })]/\text{d}~\ln \nolimits (\mathrm{\lambda })\end{eqnarray*}

Fine and coarse mode particles can be identified by calculating linear regression of ln (τ_ext_) versus ln (λ), where values close to 2 indicate fine mode and 0 represent coarse mode^51^. Absorption AE (α_abs_) can be calculated using:

(2) \begin{eqnarray*}{\mathrm{\alpha }}_{\mathrm{abs}}=-\text{d}~\ln \nolimits [{\mathrm{\tau }}_{\mathrm{abs}}(\mathrm{\lambda })]/\text{d}~\ln \nolimits (\mathrm{\lambda })\end{eqnarray*}

Aerosol particles’ physical characteristics affect α_abs_. For example, α_abs_ varies from less than 1.0 and up to 1.6 for large black carbon particles (r > 0.1 µm) based on their core size and coating structure^52^. Black carbon particles are characterized by their small-radius and spherical shapes that result in α_abs_ values of ∼1.0^53^. It is also shown that α_abs_ varies between 1.2 –3.0 for dust, 1.2 –2.0 for pollution (biomass only) and 0.75 –1.3 for mixed aerosols^54–56^.

Figures 7a, 7b, 7c and 7d show the AE over land for land region 1, over two separate band ranges over the ocean region, and over land region 2, respectively. While the spectral bands over ocean and land differ, trends across the entire region can still be followed. From mid-spring, starting at the end of March, to mid-summer, ending at the end of July, both land regions appear to have very high AE values, but the ocean values appear close to 0, implying that little is being transported across the ocean at these times. However, during other time periods AE values appear similar throughout the areas plotted. For example, peak values appear in the center of land region 1, falling in the range mentioned above for dust dominant aerosol plumes (Figures 7a).

These peaks, from November 2007 to February 2008, can be traced to the ocean domain with values falling in the mixed aerosols range, indicating that transported dust is mixed with other aerosols (Figures 7b and 7c). Furthermore, these peaks can now be tracked to land region 2, where AE values show the dominance of dust (Figure 7d). This finding confirms the fact that only very small particles, like fine dust, will be transported long range. Larger particles that mixed in with the plume along the ocean portion of the transport have mostly or completely settled.

**Figure 8.**
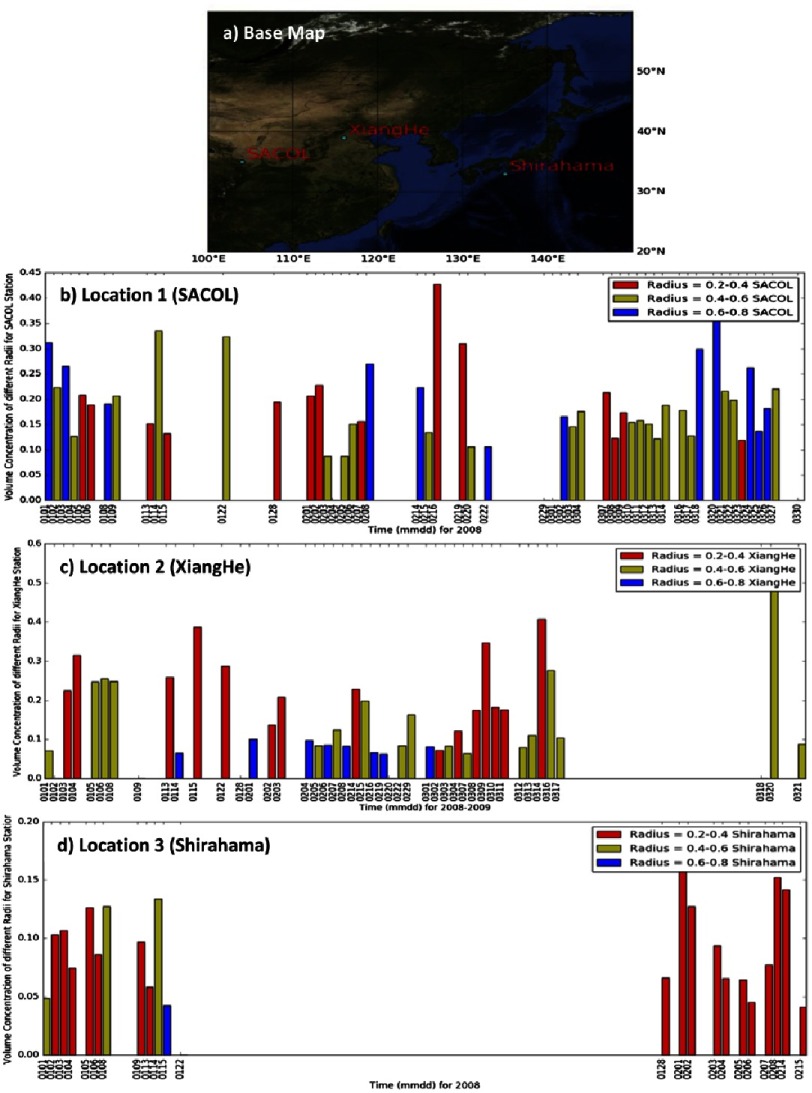
A map of the locations of the AERONET stations used and plots of retrieved volume concentration of different groupings of aerosol radii.

## Aerosol volume concentration analysis using AERONET data

Microparticle transport plays a major factor in changing aerosol types that can be investigated in a network of ground based, upward looking sun photometer (AERONET) sites^49^. In this section, we try to shed light on a possible aerosol transport between three AERONET sites, namely SACOL, Xiang He and Shirahama stations. The three sites are chosen based on their geographic locations to help track aerosol movement from China to Japan (Figure 8a). Aerosol volume size distributions over three different ranges, namely 0.2–0.4 µm, 0.4–0.6 µm and 0.6–0.8 µm, are presented to show dominant size distribution over the three AERONET sites during the period of transport.

The AERONET sun photometers are globally distributed to measure columnar spectral AOD and water vapor at a wavelength ranging from 340 nm to 1640 nm, and temporal resolution of 600 to 900 seconds. The network also retrieves columnar optical aerosol properties (e.g., aerosol size distribution, volume mean radius, volume concentration, and multi wavelength single scattering albedo at 440, 675, 870, and 1020 nm^57, 58^. This takes place by fitting measurements of the spectral AOD and sky radiances to radiative transfer calculations^54^. AERONET data retrievals comprise 1–2% estimated uncertainty^59^, with the highest uncertainty near UV wavelengths^57^.

Here, we use the AERONET Lev. 2 Ver. 2.0 products that contain retrievals for 116 different aerosol parameters including, Aerosol Volume Size Distribution (AVSD): dV(r)/dlnr (µm^3^/µm^2^) retrieved in 22 logarithmically equidistant radial bins spanning the range of particle radii 0.05 µm ≤ r ≤15 μm. Inversion products also provide the mean geometric radii of the fine and coarse modes, their standard deviation, and their volume concentrations. This is of particular importance; our goal is to understand the varying abundance of the different aerosol volume concentrations and their association with KD outbreaks^12^.

Although ground data are sometimes sparse, they are nonetheless useful when tracking long-range aerosol movement, as they help to understand different aerosol mixing scenarios over the region. The plotted data show the volume concentration of certain aerosol groupings by their radius (Figures 8b, 8c & 8d).

A possible transport event was observed from 1–2 February 2008. A large amount of fine mode aerosol particles were sampled at the SACOL station. A lesser amount of aerosols in the same radii group appeared at the Xiang He station from 2–3 February 2008. Still decreasing, a smaller amount of the same aerosol type appeared at the Shirahama station between 3–6 February 2008.

Generally evident is that the fine aerosol sizes dominate over the Japanese site, and they coincide with reported KD outbreaks. The KD data agree with findings that anthropogenic aerosols (i.e., AE > 1) are significant over the land areas of Indochina Peninsula, the Philippines and southern China, with temporal changes evident over the latter two^35^. This is in contrast to central to northern China, with AE in the range of 0.4–0.6, which is blanketed with a mixture of dust and pollution. Most ocean areas in East Asia are covered with a weak-to-moderate amount of anthropogenic aerosols, which increased significantly over the East China Sea; this was detected from MODIS aboard the Aqua satellite and was possibly due to inland pollution drifting out to sea^35^. Unfortunately, the sparsity of aerosol data that coincided with KD outbreak information meant that seasonal comparisons or additional data features could not be determined. A more complete picture is possible if more recent data are obtained.

## Aerosol vertical structure transport tracking and type information using CALIPSO

We also leveraged CALIPSO, a Franco–American satellite and space-based mission that supplies a unique data set of atmospheric vertical profiles measured by CALIOP, a Cloud-Aerosol Lidar with Orthogonal Polarization. CALIOP measures aerosol and cloud properties along with aerosol subtype information^60^. The profiles range from Earth’s surface to 40 km with a resolution of 30–60 m in the vertical and 333 m in the horizontal. CALIPSO can detect optical depths of 0.01 or less, so it can observe weak aerosol layers and thin clouds^61^.

**Figure 9.**
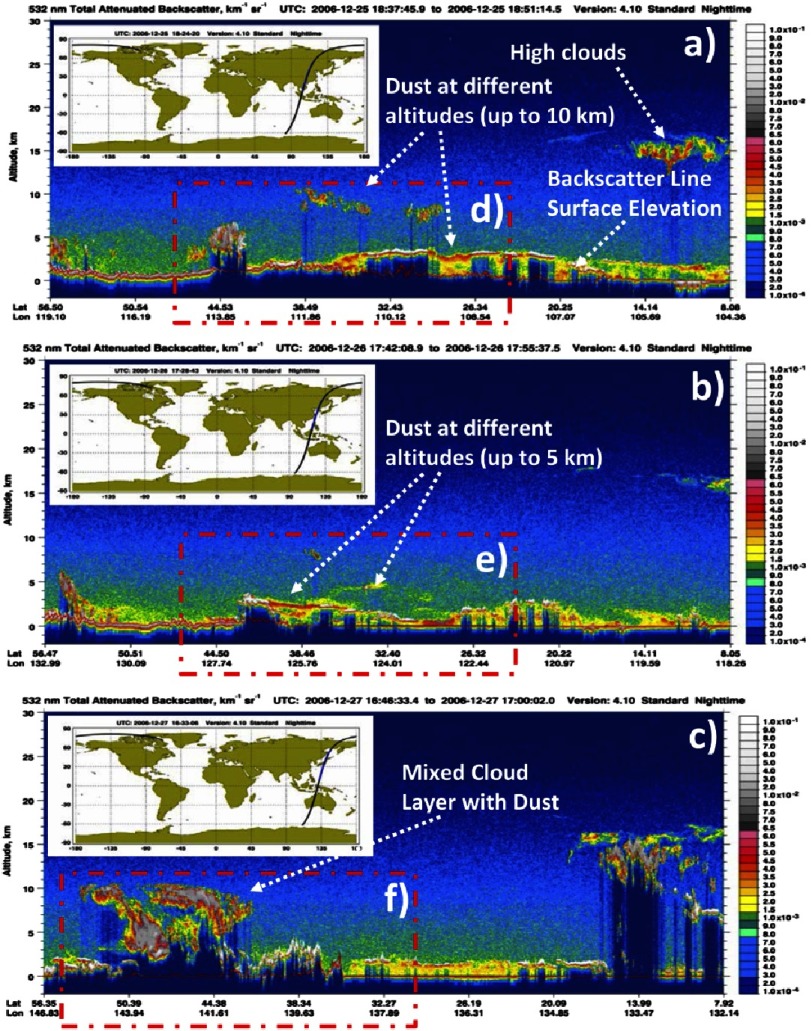

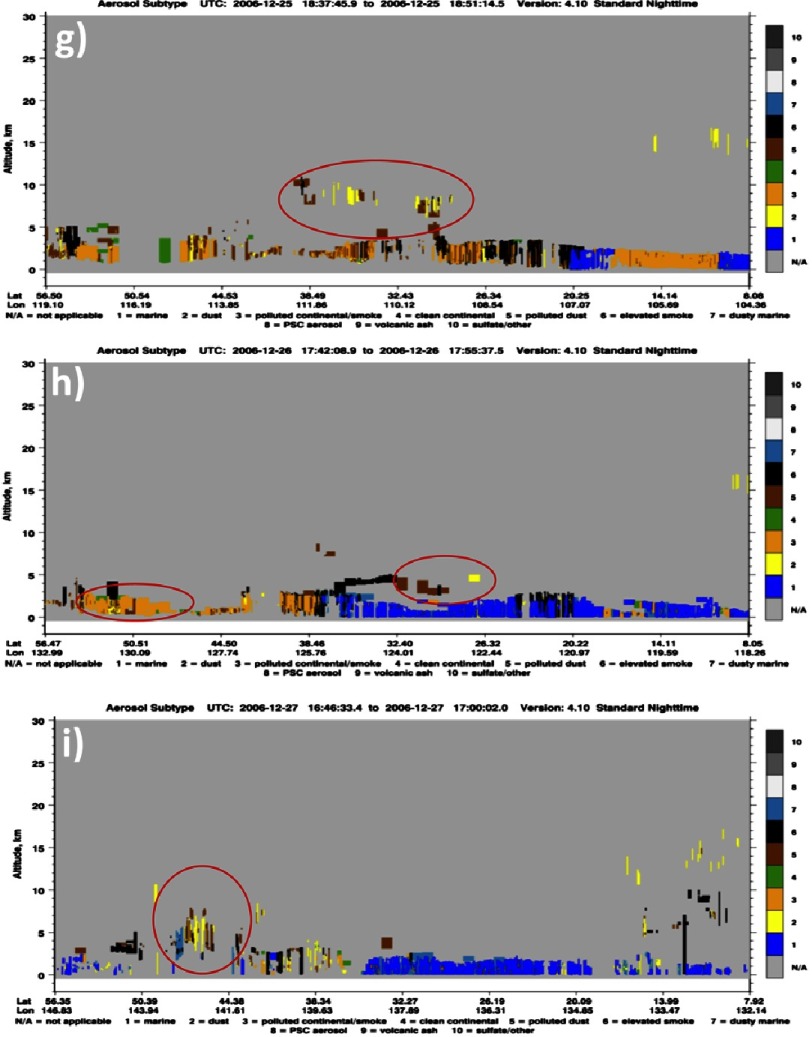
The CALIPSO attenuated backscatter of aerosol plumes moves west to east (9a–9c) with their associated orbit path and highlighted region of interest (9d–9f) and the CALIPSO aerosol subtype classifications for the same scenes (9g–9i) with highlighted areas of interest.

In this study, the CALIOP Level 1B data were employed, which contain calibrated and geo-located single-shot (highest resolution) light detection and ranging (LIDAR) profiles. Nighttime CALIOP profiles are generally better as they depict dust storms more accurately compared to daytime overpass data, which are noisier^62^. There are 10 subtypes categorized by the CALIPSO retrieval algorithm, but those of greatest interest for this study are the dust and polluted dust subtypes^63^. Figure 9 (a–i) show vertical profiles of the atmosphere up to 30 km, represented by total attenuated backscatter at 532 nm, together with the CALIPSO passes on 25, 26 and 27 December, 2006 and the most abundant aerosol types over selected areas that had been reported previously in similar studies over different polluted regions^38, 64–66^.

Using the backscatter data, aerosol plumes were tracked as they moved across the region of interest. Figure 9a shows aerosol plumes ranging from 2-12 km altitude over eastern China on December 25th (Figure 9d), in agreement with the varying size distribution (Figure 8b). Figure 9g with the region 9i, shows the aerosol subtype suggestive of plumes consisting of dust and polluted dust. On December 26th, over China, and about 15 degrees farther east, a group of aerosol plumes lost 2-5 km altitude. This follows the pattern that dust decreases in height as it is transported over a distance (Figure 9b & region 9e). The corresponding subtype analysis (Figure 9h) reveals that dust and polluted dust dominated in the plume, although this time elevated smoke appears in the region, indicating that the original plumes mixed with local aerosols. Lastly, a day later, backscatter data over Japan show a collection of aerosol plumes ranging from 1-10 km in height (Figure 9c & region f). The aerosol subtype map (Figure 9i) suggests greater heterogeneity in aerosols, yet with a definite grouping of dust and polluted dust among the lower aerosols, suggesting that this dust mixture may have been transported from the original plumes in 9a.

Since long-range transport of aerosols (including fine desert mineral dusts and biological constituents that can pose a health risk) can be followed and predicted, a global Dust-Health Early Warning System could be exploited to address KD^67^. The will to exploit this state of affairs may be aided by a recent global assessment of sand and dust storms that had been commissioned from the UN Secretary General’s office^68^.

## Conclusions

Advanced space-based technology, computing resources, environmental modeling and global monitoring networks have opened doors to solving puzzles such as the one addressed here: why and how Kawasaki disease is linked to the environment. Most important, this study makes the likelihood of predicting conditions and reducing risks for KD outbreaks possible. The research and the technology behind this study are integrated with the necessary global alliances, international collaboration of health and environment agencies and laboratories, to do just that. It is noteworthy that UN agencies recognize a myriad of windblown-dust health issues and acknowledge the scientific advances and worldwide operational weather and health services that make it possible to address them. The work reported here, measuring and following different properties of aerosol plumes, (like aerosol characteristics, plume vertical profile, and plume volume concentration), allows a well-rounded understanding of the trigger and transport scenarios for the greatest environmental suspects in KD outbreak in eastern China, Japan and possibly the western United States. We can, with certainty, locate aerosol plumes of the suspected type, such as dust, and use its signature to track and study its composition, including changes, over time. This work shows that the KD outbreak data and seasonal dust events and winds do occur in line, in sequence, over the study regions.

Where is research likely to go from here? Data examined herein contains a large amount of dimensionality – of values described in the paper as well as other parameters in each of the datasets we have discussed. Unseen correlations or patterns for clearer understanding may exist, perhaps waiting to be revealed using deep learning techniques to predict and track KD outbreaks using a greater range of geophysical and epidemiologic parameters.
